# Positive fEMG Patterns with Ambiguity in Paintings

**DOI:** 10.3389/fpsyg.2017.00785

**Published:** 2017-05-16

**Authors:** Martina Jakesch, Juergen Goller, Helmut Leder

**Affiliations:** Department of Basic Psychological Research and Research Methods, University of ViennaVienna, Austria

**Keywords:** empirical aesthetics, ambiguity, fEMG, emotion, fluency

## Abstract

Whereas ambiguity in everyday life is often negatively evaluated, it is considered key in art appreciation. In a facial EMG study, we tested whether the positive role of visual ambiguity in paintings is reflected in a continuous affective evaluation on a subtle level. We presented ambiguous (disfluent) and non-ambiguous (fluent) versions of Magritte paintings and found that *M. Zygomaticus major* activation was higher and *M. corrugator supercilii* activation was lower for ambiguous than for non-ambiguous versions. Our findings reflect a positive continuous affective evaluation to visual ambiguity in paintings over the 5 s presentation time. We claim that this finding is indirect evidence for the hypothesis that visual stimuli classified as art, evoke a safe state for indulging into experiencing ambiguity, challenging the notion that processing fluency is generally related to positive affect.

## Introduction

Imagine, you are searching for a room at a conference where you’re supposed to give a talk and you are very late. Finally, you spot a long awaited sign with two direction arrows pointing in different directions. Everyone has probably experienced the negative feeling of uncertainty arising (where to go?), frustration, or even anger in the end. Negative emotional consequences of ambiguity in everyday life situations are well documented (e.g., Hock and Krohne, 2004). Particularly in the visual domain, ambiguous information is associated with low processing fluency (Reber et al., 2004), which has been repeatedly shown not to be preferred (for an overview see Alter and Oppenheimer, 2009). These negative evaluative reactions to ambiguous, or low-fluent stimuli, are considered to be partially automatic and hard-wired and among others manifest in subtle affective reactions measurable with facial electromyography (fEMG; Topolinski et al., 2009).

Nevertheless, there are exceptions: if you see an image of the above-described ambiguous sign online, in a film, or art book, you might smile or even laugh about it. In a secure context or mode, without expectation of negative consequences, or a lack of need to act, ambiguity might have the potential of being the source of positive, pleasurable events (Gerger et al., 2014; Brieber et al., 2015). It has been shown that mere context manipulations are sufficient to elicit positive fEMG reactions for otherwise negative images (Gerger et al., 2014). Thus, it seems plausible that ambiguity elicits different reactions in an everyday context compared to an art context. Ambiguity is a central concept in art, in that some art historians even claim that artworks without ambiguous content have little artistic value at all (Krieger and Mader, 2010). However, ambiguity can take many different forms, relating to different conceptualizations on different levels. In the current study, we focus on visual ambiguity in that we use paintings showing elements which can be interpreted in different ways. We used paintings in contrast to real-world images, to ensure that the stimuli are likely to be classified as objects of art, which is an essential precondition in the process of aesthetic judgment formation (Leder et al., 2004; Leder and Nadal, 2014).

In a series of previous experiments (Jakesch et al., 2013; Jakesch and Leder, 2015), we tested whether visual ambiguity in paintings elicits higher aesthetic judgments (operationalized by interest and liking ratings). We used digital reproductions of artworks by Rene Magritte and altered versions of these in which we reduced the ambiguity which was defined as semantic or syntactic incoherence. We found that ambiguous paintings were generally rated as more interesting and pleasing. Nevertheless, at the same time, they were rated as harder to perceive and were cognitively more demanding. These findings seem to be in line with a positive role of ambiguity in art but in conflict with the processing fluency approach, where we would expect that ambiguous stimuli are liked less because of lower fluency.

However, whether the liking of visual ambiguity in paintings is an automatic, affective effect remains yet to be shown. The explicit, deliberate ratings given in our previous experiments might reflect a merely cognitive aesthetic judgment, whereas processing fluency might work on a more implicit, affective level. Both components are acknowledged in our model of aesthetic experience, where we distinguish between a cognitive state leading to an aesthetic judgment and a continuous affective evaluation leading to an aesthetic emotion (Leder et al., 2004; Leder and Nadal, 2014). Importantly, the two outcomes do not necessarily depend on each other and even can point to opposite directions. We all know situations where our “gut” and our “brain” respond differently to things we perceive. Emotionally we might shiver and feel disgust when we see a snake but we cognitively know that this snake is no harm for us in the context of a zoo. In this context, we can enjoy the beautiful color and pattern of the snakes’ skin without risking to be harmed. Such dissociation between the two outcomes was also empirically shown, in that art experts showed differences on a cognitive level compared to non-professionals, but showed similar affective reactions measured by fEMG (Leder et al., 2014). In the current study, we used fEMG as a measure for the continuous affective evaluation and liking ratings as a measure for the aesthetic judgment. The combination of these two measures enables the differentiation between two processes, which are potentially independent from each other. Facial EMG allows us to measure even subtle and implicit changes in positive and negative affect. Positive affect is associated with a higher activation of the smiling muscle, the *Musculus zygomaticus major* region and a lower activation of the frowning muscle, the *Musculus corrugator supercilii* region, whereas negative affect is associated with the opposite pattern (e.g., Larsen et al., 2003).

We tested two mutually exclusive hypotheses: (A) Visual ambiguity in paintings elicits a positive continuous affective evaluation. Thus, in comparison to real-world images, for viewing paintings the role of processing fluency on affect might be reversed. (B) Visual ambiguity in paintings elicits a negative continuous affective evaluation, despite the positive aesthetic judgments found in previous studies. This result would be in line with predictions of the processing fluency approach, in that processing fluency elicits negative affective states, on a subtle, emotional level. However, this affective component might feed into a positive cognitive evaluation (Leder et al., 2014). We expected that fluency is particularly relevant for the initial response, early after stimulus onset. This initial negative affective evaluation would then be overwritten by the aesthetic judgment at the end of each trial, which can be interpreted as a cognitive regulation. The pre-classification of an image as a piece of art facilitates “[…] an exceptional state of mind which is qualitatively different from ‘normal’ everyday mental states.” (Markovic, 2012, p. 12). Similar patterns have been found for surprising situations, which initially elicit negative affect but can then be the source for positive affect later on (Topolinski and Strack, 2015).

In the context of aesthetic experiences (Leder and Nadal, 2014) and processing fluency (Graf and Landwehr, 2015), time is an important factor in modulating different outcomes. We therefore plotted the fEMG measures over a 5 s period, in order to analyze temporal aspects. Moreover, previous research in empirical aesthetics and in ambiguity indicates that inter-individual differences influence aesthetic responses and judgments (e.g., Swami et al., 2010). In order to address these putative differences, we additionally employed a set of relevant personality scales, regarding Tolerance for Ambiguity (TAS; Herman et al., 2010), Need for Cognitive Closure (NCC; Schlink and Walther, 2007), and Personal Need for Structure (PNS; Machunsky and Meiser, 2006).

## Materials and Methods

### Participants

A total of 56 female undergraduate students (*M*_age_ = 22.05 years, *Mdn*_age_ = 21, *SD*_age_ = 3.69, age, range: 18–38) took part in exchange for course credit. All participants had normal or corrected-to-normal visual acuity, color vision, and stereopsis. This research was approved by the Ethical Committee of the University of Vienna.

### Stimuli

We used 36 ambiguous artworks by the Belgian surrealist artist Rene Magritte (see Table A1 in the Appendix for a list of names) and produced non-ambiguous versions by manipulating the artworks via Adobe Photoshop (Jakesch et al., 2013). We selected artworks depicting one specific element producing a semantic or syntactic distortion from what we usually expect in reality. For the non-ambiguous versions, we carefully replaced this unrealistic element by copying background information or any other information of the original artwork. For example, “The Explanation” (“L’explication,” 1954) shows two glass bottles, in which one bottle’s bottleneck is painted as the forepart of a carrot. For the non-ambiguous version, we replaced the carrot bottleneck by the (normal) bottleneck of the other bottle (for additional examples also see Jakesch et al., 2013). We rescaled each painting to a size of 369 pixels × 490 pixels, in which half the paintings were upright and half were landscape format. We then randomly assembled two sets of 18 ambiguous and 18 non-ambiguous paintings, in which only one version of each painting appeared in each set.

### Procedure

Participants were informed about the experimental procedure before they gave written consent. To avoid demand characteristics in collecting fEMG data, we told the participants that skin conductance responses would be recorded (e.g., Gerger et al., 2011). We then recorded their age, and tested their visual acuity, color vision, and stereopsis. Participants were comfortably seated approximately 1 m in front of a LCD monitor (NEC MultiSync 3090 WQXi, 30″, 2560 pixels × 1600 pixels) before the electrodes were applied. Participants were instructed to avoid extensive movements, chewing, and talking. They began by reading onscreen instructions and completing four practice trials before the actual experiment started. Each participant saw one set of paintings (half of them ambiguous), but two times, in two subsequent blocks. In one block, participants rated the paintings for liking, in the other block for fluency, counterbalanced across participants. Fluency was measured on a seven-point Likert scale by asking, “How easy was it for you to perceive the picture?” between 1 (*very hard*) and 7 (*very easy*). Liking was measured by asking, “How much do you like the picture?” between 1 (*not at all*) and 7 (*very much*). Each trial began with a fixation cross, presented in the center of the screen for 2 s followed by the painting for 5 s. After that, participants rated either the fluency or the liking of the painting. Each trial closed with a blank screen, presented for 3–4 s as a random inter-trial interval. After the experiment, participants rated each painting for familiarity on a seven-point Likert scale ranging from 1 (*completely unfamiliar*) to 7 (*very familiar*). Participants were instructed that these ratings refer to the familiarity of the paintings *before* this study. At the end, they filled in a computer-based post-questionnaire assessing personality factors (see Post-questionnaire).

### Facial EMG

Bipolar electrodes (Ag-AgCl, 4 mm diameter) filled with electrode gel were attached over the left side of the face covering the *M. zygomaticus major* and the *M. corrugator supercilii* regions. An electrode on the right temporal bone served as ground. The skin was rubbed with abrasive gel and cleaned with alcohol. The impedances of all electrodes were reduced below 10 kΩ (Fridlund and Cacioppo, 1986). Facial EMG was measured with a 32-channel amplifier (TMS International, Enschede, Netherlands) and sampled with 2048 Hz. The signals were filtered with a 20 Hz high-pass filter, a 500 Hz low-pass filter, a 50 Hz notch filter (to reduce power line artifacts), rectified (full-wave), and smoothed (125 ms). Additionally, videos were recorded to remove trials showing movement artifacts (Gerger et al., 2011). In doing so, between 50 and 72 (*Mdn* = 65) trials remained for further analyses. The fEMG scores represent changes in activity from the baseline, defined by the mean activity during the last second before stimulus onset. These values were then z-standardized within participants and channels. Data processing was performed with Matlab (R2014a; The MathWorks, Natick, MA, USA) using the EEGLAB toolbox (13.0.0b) and SPSS (22.0.0.1; IBM, Armonk, NY, USA).

### Post-questionnaire

In order to analyze how potentially relevant inter-individual differences affect the physiological reaction on ambiguity, we additionally measured three akin constructs: (1) TAS (Herman et al., 2010), comprising 12 items (e.g., “I can be comfortable with nearly all kinds of people”), (2) NCC (Schlink and Walther, 2007), comprising 16 items (e.g., “I don’t like unpredictable situations.”), and (3) PNS (Machunsky and Meiser, 2006) comprising 12-items (e.g., “I do not like uncertain situations.”). TAS and NCC were scored on a five-point Likert scale, PNS on a six-point Likert scale. We expected that the differences between ambiguous and non-ambiguous paintings would be more pronounced for participants showing a high tolerance for ambiguity, low cognitive need for closure, and low personal need for structure, respectively.

## Results

The overall familiarity of the pictures was rather low, with a mean value of *M* = 2.61 (*SD* = 0.62) on a seven point-scale ranging from 1 (*completely unfamiliar*) to 7 (*very familiar*). Importantly, the familiarity ratings of the ambiguous paintings (*M* = 2.53, *SD* = 0.66) did not significantly differ from the non-ambiguous versions (*M* = 2.68, *SD* = 0.59), statistically confirmed by running a *t*-test for independent groups, *t*(70) = 1.02, *p* = 0.312.

### Facial EMG Data

**FIGURE 1:**
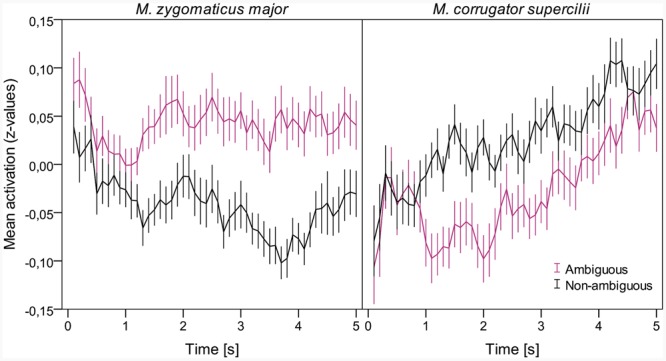
**Time course of the z-standardized (within-participant) activation of the *M. corrugator supercilii* and *M. zygomaticus* major regions, plotted separately for ambiguous and non-ambiguous artworks.** Error bars show standard errors.

**Figure 1** shows the time course of both muscle regions over the 5 s presentation time (cut into 100 ms segments) sampled over all participants and both blocks. We found very similar patterns for the liking and the fluency blocks, which is why we do not report them separately. Collapsed over ambiguous and non-ambiguous paintings, *M. zygomaticus major* showed a relatively straight activation compared to the base rate, whereas *M. corrugator supercilii* activation slowly increased over time. Importantly for our hypotheses, both graphs show a difference in the activation between ambiguous and non-ambiguous paintings, slightly different for both muscle regions. *M. zygomaticus major* showed a relatively homogenous pattern over time, which reached its maximum difference after about 3–4 s after stimulus onset. For *M. corrugator supercilii*, the pattern showed a divergence between ambiguous and non-ambiguous paintings after about 1 s after stimulus onset, before the difference slowly declined over time. The main difference for ambiguity was confirmed by running two repeated measures *t*-tests with ambiguity as within-participant factor (ambiguous, non-ambiguous) and the z-standardized fEMG scores as dependent variables. Ambiguous paintings evoked higher *M. zygomaticus major* activation (*M* = 0.042, *SD* = 0.094) than non-ambiguous paintings (*M* = -0.042, *SD* = 0.091), *t*(55) = 3.4, *p* = 0.001, Cohen’s *d* = 0.91. The opposite pattern was found for *M. corrugator supercilii*, where ambiguous paintings evoked lower activation (*M* = -0.028, *SD* = 0.081) than non-ambiguous paintings (*M* = 0.027, *SD* = 0.08), *t*(55) = 2.57, *p* = 0.013, Cohen’s *d* = 0.69. In order to additionally control for the variation between the different paintings and estimate interactions with time, we ran two linear mixed models (LMMs) for both muscle regions, applying Satterthwaite approximation for *p*-values. One advantage of applying LMMs is to include the stimulus variation in the statistical model and therefore control for potential artifacts merely caused by stimulus selection. We included a successive-difference-coded contrast for fixed effects of ambiguity (ambiguous minus non-ambiguous) and time as a continuous, centered fixed effect (with a temporal resolution of 100 ms). We also included the interaction between the two fixed effects and random by-artwork and by-participant intercepts. We did not include random slopes as otherwise the models failed to converge. For *M. zygomaticus major*, we found a significant effect of ambiguity, *b* = 0.093, *SE* = 0.026, *t*(70) = 3.61, *p* < 0.001, a significant effect of time, *b* < -0.001, *SE* < 0.001, *t*(181,600) = -3.69, *p* < 0.001, and a significant interaction, *b* < 0.001, *SE* < 0.001, *t*(181,600) = 4.32, *p* < 0.001. For *M. corrugator supercilii*, we found an effect by trend of ambiguity, *b* = -0.048, *SE* = 0.025, *t*(70) = 1.92, *p* = 0.0585, a significant effect of time, *b* < 0.001, *SE* < 0.001, *t*(181,600) = 16.8, *p* < 0.001, but no significant interaction, *b* < 0.001, *SE* < 0.001, *t*(181,600) = 0.86, *p* = 0.388.

### Personality Scales

We additionally tested whether the difference in the muscle activation was moderated by personality factors. For each participant, we calculated a raw score for each scale. The mean value for TAS was, *M* = 42.5 (*SD* = 5.21, *Mdn* = 42.5, range: 30–53), for NCC, *M* = 50.98 (*SD* = 10.05, *Mdn* = 51.5, range: 26–71), and for PNS, *M* = 42.14 (*SD* = 6.96, *Mdn* = 43, range: 19–57). The scales were moderately correlated (all *p*s < 0.001; *r*s between *r* = 0.49 and *r* = 0.69). We used the median of each scale to split the sample into two groups, before we ran three mixed-design ANOVAs with ambiguity as within-participant factor and the median splits as between-participants factors with muscle activation as dependent variable. For *M. corrugator supercilii*, each ANOVA showed a main effect for ambiguity, *F*(1,54) ≥ 6.5, *p* ≤ 0.014, but no main effects for the scales, *F*s(1,54) ≤ 2.03, *p*s ≥ 0.16, and no significant interactions, *F*(1,54) ≤ 0.92, *p* ≥ 0.34. For *M. zygomaticus major*, the same pattern was found, with each ANOVA showing a main effect for ambiguity, *F*(1,54) ≥ 11.33, *p* ≤ 0.001, but no main effects for the scales, *F*(1,54) ≤ 0.56, *p* ≥ 0.46, and no significant interactions, *F*(1,54) ≤ 1.17, *p* ≥ 0.29. We also found no significant correlation between the differences in fEMG scores between ambiguous and non-ambiguous paintings and the scales’ raw scores, *r*s ≤ 0.12, *p*s ≥ 0.39.

### Fluency and Liking

As expected and in line with previous findings (Jakesch et al., 2013), the fluency ratings were significantly lower for ambiguous paintings (*M* = 4.98, *SD* = 0.84) than for non-ambiguous paintings (*M* = 5.84, *SD* = 0.66), statistically confirmed by a repeated-measures *t*-test, *t*(55) = 10.38, *p* < 0.001, Cohen’s *d* = 1.14. However, there was no significant difference in liking ratings between ambiguous (*M* = 3.97, *SD* = 0.76) and non-ambiguous paintings (*M* = 3.89, *SD* = 0.86), *t*(55) = 0.76, *p* = 0.45, Cohen’s *d* = 0.10. To further analyze the lack of a main effect for liking, we added the TAS, NCC, and PNS as covariates as well as between-participants factors in further ANOVAs. However, we found no significant interactions between ambiguity and any of the scales. We additionally analyzed only those liking ratings, which were given in the first block, to eliminate possible sequential effects. Ambiguous paintings were slightly liked more (*M* = 3.91, *SD* = 0.62) than their non-ambiguous counterparts (*M* = 3.70, *SD* = 0.82) in the first block, although the difference was also not significant, *t*(27) = 1.56, *p* = 0.13, Cohen’s *d* = 0.28. Looking closer at the differences on an individual level, we found that 22 out of 56 participants (39%) liked the non-ambiguous versions more, putatively diminishing the main effect for liking. We also calculated the individual differences in liking between ambiguous and non-ambiguous paintings for each participant to compare them with the differences in muscle activation. We found no significant correlations between the differences in liking and the differences in the *M. corrugator supercilii* activation, *r* = -0.078, *p* = 0.57, or the *M. zygomaticus major* activation, *r* = 0.061, *p* = 0.66.

## Discussion

We studied the effects of visual ambiguity on aesthetic judgments and continuous affective evaluations in aesthetic experiences (Leder and Nadal, 2014). We presented ambiguous and non-ambiguous versions of Magritte paintings and measured fEMG patterns over the 5 s presentation time. We thereby tested whether visual ambiguity in paintings elicits (A) a positive or (B) a negative continuous affective evaluation. Our results support hypothesis A in that ambiguous painting elicited a significant higher *M. zygomaticus major* activation and a lower *M. corrugator supercilii* activation than their non-ambiguous counterparts did. This pattern of facial muscle activation is generally associated with positive affective and emotional reactions (Larsen et al., 2003). **Figure 1** gives an idea of the temporal development of this effect. It seems that the difference in ambiguity emerges early after stimulus onset for both muscle regions. For *M. zygomaticus major*, this initial difference further increases over time and reaches its maximum after about 3–4 s. The interaction between ambiguity and time statistically supports this impression. This effect might also indicate that the positive effect of ambiguity on the continuous affective evaluation needs time to fully unfold. For *M. corrugator supercilii*, the largest difference occurs after about 1–2 s, before the difference slowly declines over time. Together, our main findings are in line with art-historical approaches (e.g., Krieger and Mader, 2010) and previous empirical findings (Jakesch et al., 2013; Jakesch and Leder, 2015; Muth et al., 2015), which claim that not the (full) resolution of ambiguity seems to be the source of positive responses but rather the resolvement itself.

Particularly the initial positivity in the fEMG pattern for ambiguous paintings contradict what the processing fluency approach would have predicted (Topolinski et al., 2009). In fluency research, it is often claimed that higher fluency in visual perception automatically elicits a positive response. As shown in this study, this automaticity seems to be reversed for paintings, compared to everyday-life stimuli. This challenges the generalization of the automaticity of the fluency approach, in that its function can be overwritten by top-down processes. People might have learned that the possibility is significantly higher to be confronted with uncertain, ambiguous content in paintings than in real world scenes. This knowledge might provide a safe context and evoke an inner state to playfully deal with ambiguity (Dissanayake, 2007) without any negative consequences. This distance to the stimulus might then be the reason why we can enjoy negative, ambiguous, or even horrifying content in an art context (Cupchik, 2002; Gerger et al., 2014). Our findings further highlight the importance of stimulus classification in visual processing. We explicitly informed our participants that the images they saw were works of art. It thereby might be that such a pre-classification is a necessary precondition for showing positive affective responses to ambiguity. Future studies could also focus on affective responses to paintings in surprise paradigms to test what preconditions are sufficient to show positive responses to ambiguous paintings.

We further compared the fEMG patterns with liking ratings, representing an explicit aesthetic judgment (Leder and Nadal, 2014). In previous, behavioral studies, we repeatedly found that ambiguous paintings were liked more than non-ambiguous paintings (Jakesch et al., 2013; Jakesch and Leder, 2015). However, although we used the same stimuli, we found no main effect for liking in the current study. A closer look on an individual level reveals that in all our previous experiments 25–29% of our participants showed the opposite pattern, in that they liked the non-ambiguous paintings more. In the current study, however, this number rose to 39%, dissolving the main effect for liking. The most parsimonious explanation for why we are not able to report significant main effects for liking might be that we simply by chance sampled more participants, who generally like ambiguous paintings less. However, we think that it is more likely that two differences in the study design were decisive. First, our participants were attached with electrodes and were told that their skin conductance would be measured. Such a setting possibly affects the expectations and the assumptions of participants in a systematic way. Although we afterward asked our participants if they had hypotheses in mind during the testing, we did not specifically ask, how the setting influenced their ratings. A more detailed post-questionnaire in future studies might help to get a more comprehensive insight. Second, in our behavioral studies, the paintings were presented at longest for 1 s, whereas here we presented them for 5 s. The longer presentation time might have made the ambiguity in the paintings appear less interesting and therefore liked less. However, the divergence between the fEMG pattern and the behavioral ratings might suggest that the continuous affective evaluation and the aesthetic judgment not necessarily have to be aligned (Leder et al., 2004; Leder and Nadal, 2014). Thus, the fEMG patterns over time might reflect a different evaluative process than the explicit ratings given at the end of the trial.

Although the lowered activation of the *M. corrugator supercilii* region in our study is often associated with negative affective and emotional reactions (Larsen et al., 2003), there is also room for alternative explanations. We know that *M. corrugator supercilii* activity can also be associated with high cognitive load and certain positive emotions, like surprise (Topolinski and Strack, 2015). For both alternatives, we would expect the reversed pattern for ambiguous paintings than that which was actually found. If cognitive load or surprise was driving the *M. corrugator supercilii* activation, it should have been higher for ambiguous paintings. Another reason to be cautious is the lack of a significant main effect (*p* = 0.059) for ambiguity on the *M. corrugator supercilii* region in the LMM. We see in **Figure 1** that the main effect varies over time in a non-linear fashion. This might be the reason why we found no main effect for ambiguity and no interaction between ambiguity and time for the *M. corrugator supercilii* region.

Moreover, none of the three personality scales, TAS (Herman et al., 2010), NCC (Schlink and Walther, 2007), and PNS (Machunsky and Meiser, 2006) showed any effect on the fEMG scores or behavioral measures. Previous experiments testing ambiguity also showed only a moderate influence of TAS scores on aesthetic experiences (Muth et al., 2015) which might be due to the fact that all three scales were developed with a high emphasis on social situations (Machunsky and Meiser, 2006; Schlink and Walther, 2007; Herman et al., 2010). The items therefore might not be selective for our aims (see also example items in the Post-questionnaire section). Furthermore, the sum scores for the personality scales ranged in the middle of possible scores, so future studies could use a pre-screening tool in order to compare people with more diverging scores.

To sum up, we found that visual ambiguity in paintings leads to a positive continuous affective evaluation over time. This finding limits the automaticity and generalizability of processing fluency in the visual domain. As opposed to everyday perception, stimuli classified as art can lead to a positive experience despite being perceived as less fluent. This experience is not necessarily reflected on an explicit level of aesthetic judgments.

## Ethics Statement

This study was carried out in accordance with the recommendations of the Ethical Committee of the University of Vienna with written informed consent from all subjects. All subjects gave written informed consent in accordance with the Declaration of Helsinki. The protocol was approved by the Ethical Committee of the University of Vienna.

## Author Contributions

MJ made substantial contributions to the conception and design of the work, data acquisition, interpretation of data for the work, and drafting the work. JG made substantial contributions to the analysis, interpretation of data for the work, and drafting the work. HL made substantial contributions to the conception and design of the work, interpretation of data for the work, and revising it critically for important intellectual content. MJ, JG, and HL give their final approval of the version to be published and agree to be accountable for all aspects of the work in ensuring that questions related to the accuracy or integrity of any part of the work are appropriately investigated and resolved.

## Conflict of Interest Statement

The authors declare that the research was conducted in the absence of any commercial or financial relationships that could be construed as a potential conflict of interest.
