# Supplementary material for: Positive fEMG Patterns with Ambiguity in Paintings
Source: Front Psychol. 2017 May 16;8:785. doi: 10.3389/fpsyg.2017.00785 (PMC5432603; doi:10.3389/fpsyg.2017.00785)
Supplement: Supplementary file 1 [file Data_Sheet_1.docx]

# Appendix

Table A1

*List of Magritte Paintings Used for this Study*

| Original name | Year |
| --- | --- |
| Découverte | 1927 |
| La clef de verre | 1959 |
| La condition humaine | 1948 |
| La corde sensible | 1960 |
| La Décalcomanie | 1966 |
| La durée poignardée | 1938 |
| La Géante | 1929/30 |
| La grande guerre | 1964 |
| La Lampe philosophique | 1936 |
| La lunette d'approche | 1963 |
| La Magie Noire | 1933/34 |
| La main heureuse | 1953 |
| La philosophie dans le boudoir | 1965 |
| La reconnaissance infinite | 1963 |
| La reproduction interdite | 1937 |
| L'Ami de l'Ordre | 1964 |
| Le banquet | 1958 |
| Le château des Pyrénées | 1962 |
| Le dernier cri | 1967 |
| Le Domaine d'Arnheim | 1938 |
| Le faux miroir | 1935 |
| Le Modele rouge | 1947 |
| Le Mois des Vendanges | 1959 |
| Le parfum de l'abîme | 1928 |
| Le Reveille-matin | 1953 |
| Le Rossignol | 1962 |
| Le sorcier | 1952 |
| Les Ombres | 1966 |
| Les travaux d’Alexandre | 1958 |
| Les vacances de Hegel | 1958 |
| L'Esprit de géométrie | 1937 |
| L'explication | 1954 |
| L'Ile au Trésor | 1942 |
| Tentative de L'Impossible | 1928 |

*Note.* All paintings are oil paintings made on canvas
